# Exploratory analyses assessing the impact of early tumour shrinkage and depth of response on survival outcomes in patients with *RAS* wild-type metastatic colorectal cancer receiving treatment in three randomised panitumumab trials

**DOI:** 10.1007/s00432-017-2534-z

**Published:** 2017-10-28

**Authors:** Julien Taieb, Fernando Rivera, Salvatore Siena, Meinolf Karthaus, Manuel Valladares-Ayerbes, Javier Gallego, Michael Geissler, Reija Koukakis, Gaston Demonty, Marc Peeters

**Affiliations:** 1 Department of Hepatogastroenterology and GI Oncology, Georges Pompidou European Hospital and Sorbonne Paris Cité / Université Paris Descartes, 20 rue Leblanc, 75015 Paris, France; 2 Hospital Universitario Marqués de Valdecilla, Santander, Spain; 3 Grande Ospedale Metropolitano Niguarda and Dipartimento di Oncologia e Emato-Oncologia, Niguarda Cancer Center, Università degli Studi di Milano, Milan, Italy; 4 Städtisches Klinikum München, Klinikum Neuperlach, Munich, Germany; 5 Virgen del Rocio Hospital, Seville, Spain; 6 Hospital General Universitario de Elche, Elche, Spain; 7 Klinikum Esslingen, Esslingen, Germany; 8 Biostatistics, Amgen Ltd, Uxbridge, UK; 9 Amgen (Europe) GmbH, Medical Development, Zug, Switzerland; 10 Antwerp University Hospital, Antwerp, Belgium

**Keywords:** Depth of response, Early tumour shrinkage, Metastatic colorectal cancer, Panitumumab, Survival

## Abstract

**Purpose:**

To report exploratory analyses of early tumour shrinkage (ETS) and depth of response (DpR) in patients with *RAS* wild-type (WT) metastatic colorectal cancer (mCRC), receiving the first-line treatment in three randomised panitumumab trials.

**Methods:**

Data from the PRIME (NCT00364013), PEAK (NCT00819780) and PLANET (NCT00885885) studies were included. Median DpR, the proportion of patients achieving ETS ≥ 20% or ≥ 30% at week 8, and the impact of ETS and DpR (including by category) on outcome were analysed. Factors associated with ETS and DpR and the optimal ETS/DpR cut-off values for predicting improved overall survival (OS) were assessed.

**Results:**

Overall, 505, 170 and 53 patients had *RAS* WT mCRC in PRIME, PEAK and PLANET, respectively. Patients receiving panitumumab had higher ETS rates (≥ 30%: PRIME 59% vs. 38%; PEAK 64% vs. 45%) and greater DpR (PRIME: 54% vs. 46%; PEAK: 65% vs. 46%) than those receiving treatment without panitumumab. In multiple regression analyses, panitumumab treatment, liver-only metastases and WT *BRAF* status were consistently associated with improved ETS and DpR outcomes. Irrespective of treatment, ETS and DpR were associated with improved progression-free survival, overall survival and resection rates; most resections occurred in patients in the two highest DpR categories. In PRIME and PEAK, respectively, the optimal cut-offs for predicting improved OS were 32 and 34% for ETS, and 59 and 70% for DpR.

**Conclusions:**

These exploratory analyses suggest that panitumumab is associated ETS and DpR benefits in patients with *RAS* WT mCRC and that achieving these endpoints during first-line treatment is linked with favourable outcomes.

**Electronic supplementary material:**

The online version of this article (doi:10.1007/s00432-017-2534-z) contains supplementary material, which is available to authorized users.

## Introduction

Tumour response, as defined by Response Evaluation Criteria In Solid Tumours (RECIST), is a common endpoint in clinical trials, and requires that tumour shrinkage of ≥ 30% is confirmed at consecutive visits (Therasse et al. 2000; Eisenhauer et al. 2009). However, RECIST does not consider timing, depth or duration of response. Achieving early and sustained tumour shrinkage is an important treatment goal in patients with metastatic colorectal cancer (mCRC) as it may increase the chance of surgical resection and provide relief of tumour-related symptoms (Folprecht et al. 2010; Douillard et al. 2015). Two other shrinkage-related endpoints that provide information over and above that provided by RECIST have also started to be utilised in mCRC trials. Early tumour shrinkage (ETS of ≥ 20% or ≥ 30% assessed after 6 or 8 weeks of treatment) can provide an early indication of sensitivity to treatment (Piessevaux et al. 2013; Giessen et al. 2013; Modest et al. 2013; Douillard et al. 2015; Heinemann et al. 2015; Cremolini et al. 2015), whereas depth of response (DpR) assesses the maximum tumour shrinkage achieved by a patient during treatment (Heinemann et al. 2015).

In first-line trials comparing epidermal growth factor receptor inhibitors (EGFRIs) plus chemotherapy vs. bevacizumab plus chemotherapy in patients with mCRC, the EGFRIs resulted in higher rates of ETS and were also associated with greater median DpR (Stintzing et al. 2016; Rivera et al. 2017). Furthermore, exploratory analyses of first-line trial data demonstrated that both ETS and DpR were associated with improved overall survival (OS) (Mansmann et al. 2013; Cremolini et al. 2015; Douillard et al. 2015; Heinemann et al. 2015; Stintzing et al. 2016; Rivera et al. 2017). Here, we aim to consolidate the available data on the effects of panitumumab on ETS and DpR in first-line *RAS* wild-type (WT) mCRC, some of which have only been published in congress abstracts to date (Abad et al. 2014; Abad et al. 2015; Rivera et al. 2016; Siena et al. 2016). We further build on these data by reporting new exploratory analyses of the optimal ETS and DpR cut-offs to predict improved OS, multiple regression analyses of factors associated with ETS and DpR, the impact of DpR by category on outcome in PEAK, and the impact of ETS and DpR on response and resection outcomes (where available).

## Methods

### Included studies and patients

Three first-line panitumumab mCRC studies were included in these analyses. PRIME (NCT00364013) was a phase III trial comparing panitumumab plus FOLFOX4 vs. FOLFOX4 alone (Douillard et al. 2010, 2015). PEAK (NCT00819780) was a phase II study comparing panitumumab plus modified (m)FOLFOX6 vs. bevacizumab plus mFOLFOX6 (Schwartzberg et al. 2014; Rivera et al. 2017). PLANET (NCT00885885) was a phase II study comparing first-line panitumumab plus FOLFOX4 vs. panitumumab plus FOLFIRI (Abad et al. 2014, 2015).

The present analyses included data from patients in these studies who had *RAS* WT mCRC (i.e. those whose tumours contained no mutations in *KRAS* and *NRAS* exons 2 [codons 12 and 13], 3 [codons 59 and 61] and 4 [codons 117 and 146]). All analyses and *p* values are descriptive.

### Early tumour shrinkage analyses

Tumour shrinkage measurements were based on the sum of the longest diameters (mm) of measurable target lesions. *RAS* WT data were analysed to determine the proportion of patients achieving ETS ≥ 20% or ≥ 30% at week 8 (compared with baseline) in each study and the impact of ETS ≥ 20% and ≥ 30% (overall and by treatment) on progression-free survival (PFS) and OS were also assessed. Multiple regression analyses were performed to determine baseline factors associated with ETS in the PRIME and PEAK studies. ETS was included as a continuous variable (i.e. each patient’s percentage shrinkage at week 8) and a stepwise model building procedure was used, with a 10% significance level for a covariate to enter or remain in the model. The effect of ETS on RECIST response and the proportions of patients undergoing resection who experienced ETS were also evaluated, where possible.

A study-level meta-analysis was conducted to estimate the effect of ETS ≥ 20% vs. < 20% and ETS ≥ 30% vs. < 30% on PFS, OS and resection (complete [R0] and/or partial [R1] resection) rates in patients with *RAS* WT mCRC receiving first-line treatment (overall) in these three studies. Meta-analysis techniques, including fixed-effect modelling (unconditional maximum likelihood method) and random-effect modelling (DerSimonian and Laird modelling methods) (DerSimonian and Laird 1986), were used to pool study-level trial data using the inverse-variance of each study as the weight. An exploratory analysis to estimate the optimal ETS cut-off value for prediction of improved OS in the PRIME and PEAK studies was performed according to a previously published method (Contal and O’Quigley 1999).

### Depth of response analyses

DpR was calculated as the maximum percentage change from baseline to nadir in patients who had tumour shrinkage and median DpR was calculated by treatment in the three studies. DpR had a positive value for tumour reduction, negative for tumour growth, and zero for no change. Patients who had measurable disease at baseline and calculable DpR post-baseline, were included in these analyses. Multiple regression analyses were performed to determine baseline factors associated with DpR in the PRIME and PEAK studies. DpR was included as a continuous variable (i.e. each patient’s maximum percentage shrinkage) and a stepwise model building procedure was used, with a 10% significance level for a covariate to enter or remain in the model.

DpR was also analysed by category in the PRIME and PEAK studies (data not available for PLANET). Here, patients with tumour growth were categorised as having DpR < 0%, with the remainder subdivided into four additional DpR categories based on the extent of observed shrinkage. These categories included the RECIST cut-off for a partial response (30%) and three further approximately equally sized groups based on patient quartiles (PRIME DpR cut-offs: 0–30%, 31–52%, 53–70%, 71–100%; PEAK DpR cut-offs: 0–30%, 31–53%, 54–82% and 83–100%). The overall impact of DpR (irrespective of treatment) on PFS and OS outcomes and RECIST response, duration of response (DoR) and resection rates, was evaluated, with DpR evaluated both as a continuous and ordinal variable, in simple and multiple Cox regression models. The multiple Cox regression model also included terms for treatment and stratification factors (baseline Eastern Cooperative Oncology Group [ECOG] performance status and region). Exploratory analyses comparing PFS and OS in patients with DpR of ≥ 30% vs. DpR of < 30% (i.e. utilising the RECIST cut-off for response) and DpR of ≥ 20% vs. DpR of < 20%, were also performed. An exploratory analysis to estimate the optimal DpR cut-off value for prediction of improved OS in the PRIME and PEAK studies was performed according to a previously published method (Contal and O’Quigley 1999).

## Results

### Patients

Overall, 505, 170 and 53 patients had *RAS* WT mCRC in the PRIME, PEAK and PLANET studies, respectively (Table 1). Baseline demographics were generally similar between studies except that more patients were male in PLANET (77%) than PRIME (65%) or PEAK (67%). Furthermore, all patients in PLANET had liver-limited metastatic disease (in line with the study inclusion criteria), compared with 18 and 26% in the PRIME and PEAK studies, respectively.

**Table 1:** Baseline demographics and disease characteristics for patients in the PEAK, PLANET and PRIME studies with available data (*RAS* wild-type population)

	PEAK (*n* = 170)^a^		PLANET (*n* = 53)^b^		PRIME (*n* = 505)^c^	
	Pmab + FX6 (*n* = 88)	Beva + FX6 (*n* = 82)	Pmab + FX4 (*n* = 27)	Pmab + FI (*n* = 26)	Pmab + FX4 (*n* = 253)	FX4 (*n* = 252)
Male sex, *n* (%)	58 (66)	56 (68)	23 (85)	18 (69)	170 (67)	158 (63)
Age—median, years (range)	62 (23–82)	60 (39–82)	66 (32–79)	60 (37–78)	61 (27–81)	61 (24–82)
ECOG PS 0/1, *n* (%)	88 (100)	81 (99)^d^	NA	NA	238 (94)	235 (93)
Primary cancer diagnosis, *n* (%)						
Colon	64 (73)	57 (70)	NA	NA	165 (65)	164 (65)
Rectal	24 (27)	25 (30)	NA	NA	88 (35)	88 (35)
Side of disease, *n* (%)						
Left	53 (60)	54 (66)	NA	NA	169 (67)	159 (63)
Right	22 (25)	14 (17)	NA	NA	39 (15)	49 (19)
Unknown	13 (15)	14 (17)	NA	NA	45 (18)	44 (17)
Sites of metastases, *n* (%)						
Liver only	23 (26)	22 (27)	27 (100)	26 (100)	48 (19)	41 (16)
Liver + other	43 (49)	34 (41)	0 (0)	0 (0)	169 (67)	172 (68)
Other only	22 (25)	26 (32)	0 (0)	0 (0)	36 (14)	39 (15)

*ECOG PS* Eastern Cooperative Oncology Group performance status, *FI* FOLFIRI, *FX4* FOLFOX4, *FX6* mFOLFOX6, *NA* not available

^a^ *n* = 154; ^b^ *n* = 47; ^c^ *n* = 440 included in the early tumour shrinkage analyses, respectively, from these studies

^d^ECOG PS was missing/unknown for 1 patient in this group

In PRIME and PEAK, baseline demographics and disease characteristics were generally similar across DpR categories, although patients with DpR < 0% more commonly had *BRAF* mutant tumours (Supplementary Table S1). In PRIME, DpR < 0% more commonly occurred in patients receiving FOLFOX4 alone (Supplementary Table S1A), while in PEAK the three patients with DpR < 0% all received panitumumab plus mFOLFOX6 (Supplementary Table S1B). Overall, in these two studies, the three lowest DpR categories generally included proportionally more patients with right-sided tumours than the two highest DpR categories.

### Early tumour shrinkage: individual study data

#### PRIME

Overall, 440 patients were included in the ETS analyses; 283 (64%) achieved ETS ≥ 20% and 213 (48%) achieved ETS ≥ 30% (Douillard et al. 2015). Of the patients with ETS ≥ 20% and ETS ≥ 30%, respectively, 225 (80%) and 185 (87%) were subsequently confirmed as achieving a RECIST response (partial or complete), with the remainder having a best overall response of stable (SD) or progressive disease (PD).

Sixty-one patients underwent a resection (R0 and/or R1) and also had ETS data. Of these, 51 (84%) experienced ETS ≥ 20% and 42 (69%) had ETS ≥ 30%. Likewise, 44 patients had R0 resections and ETS data. Of these, 38 (86%) experienced ETS ≥ 20% and 33 (75%) had ETS ≥ 30%.

More patients receiving panitumumab plus FOLFOX4 vs. FOLFOX4 alone had ETS ≥ 20% (72% vs. 57%, odds ratio [OR]: 1.99 [95% CI 1.34, 2.96]; *p* < 0.001) or ≥ 30% (59% vs. 38%, OR 2.43 [95% CI 1.66, 3.56]; *p* < 0.001) (Douillard et al. 2015). Factors associated with improved ETS in the final multiple regression model were panitumumab treatment (vs. FOLFOX4 alone), liver-only metastases (vs. liver + other or other only metastases) and WT *BRAF* status (vs. mutant) (Table 2a).

**Table 2:** Baseline factors associated with early tumour shrinkage and depth of response (a, PRIME; b, PEAK studies) (*RAS* wild-type population; multiple regression analyses including early tumour shrinkage and depth of response as continuous variables)

(a)* Factors associated with early tumour shrinkage*	Estimate (95% CI)
Treatment	
Panitumumab + FOLFOX4: FOLFOX4	9.62 (5.7, 13.5)
Sites of metastases	
Liver + other: liver only	− 5.79 (− 11.0, − 0.60)
Other only: liver only	− 12.86 (− 19.8, − 5.9)
*BRAF* status	
Mutant: wild-type	− 10.80 (− 17.1, 4.5)
* Factors associated with depth of response*	Estimate (95% CI)
Treatment	
Panitumumab + FOLFOX4: FOLFOX4	8.16 (2.1, 14.2)
Sites of metastases	
Liver + other: liver only	− 18.26 (− 26.2, − 10.3)
Other only: liver only	− 29.13 (− 40.0, − 18.2)
*BRAF* status	
Mutant: wild-type	− 30.81 (− 40.6, − 21.0)
Eastern Cooperative Oncology Group performance status	
2: 0 or 1	− 14.39 (− 27.9, − 0.9)

(b)* Factors associated with early tumour shrinkage*	Estimate (95% CI)
Treatment	
Panitumumab + mFOLFOX6: bevacizumab + mFOLFOX6	6.73 (1.4, 12.1)
Sites of metastases	
Liver + other: liver only	0.35 (− 6.1, 6.8)
Other only: liver only	− 6.5 (− 13.6, 0.6)
*BRAF* status	
Mutant: wild-type	− 10.5 (− 20.9, − 0.2)
*Factors associated with depth of response*	Estimate (95% CI)
Treatment	
Panitumumab + mFOLFOX6: bevacizumab + mFOLFOX6	12.30 (2.9, 21.7)
Sites of metastases	
Liver + other: liver only	− 9.58 (− 20.9, 1.7)
Other only: liver only	− 19.55 (− 32.0, 7.1)
*BRAF* status	
Mutant: wild-type	− 14.78 (− 31.9, 2.3)
Age	
Continuous variable	− 0.47 (− 1.0, 0.1)

A stepwise model building procedure was used with a 10% significance level for a covariate to enter or remain in the model. Positive estimates indicate increased depth of response for the second level of the covariate relative to the first level of the covariate (level 1: level 0). Negative estimates indicate decreased depth of response for the second level of covariate relative to the first level of the covariate (level 1: level 0)

*CI* confidence interval

Amongst patients achieving ETS ≥ 30%, those receiving panitumumab plus FOLFOX4 had longer median PFS (14.9 vs. 10.9 months, hazard ratio [HR]: 0.70 [95% confidence interval {CI} 0.51, 0.94]; *p* = 0.019) compared with those receiving FOLFOX4 alone; median OS was 34.5 vs. 30.7 months, respectively (HR 0.85 [95% CI 0.62, 17]; *p* = 0.31) (Douillard et al. 2015). PFS (9.3 vs. 7.0 months, HR 0.78 [95% CI 0.59, 1.03]; *p* = 0.790) and OS (18.2 vs. 16.0 months, HR 0.80 [95% CI 0.60, 1.06]; *p* = 0.1249) outcomes were similar between treatments for those patients with ETS < 30%. Irrespective of treatment received, patients achieving ETS ≥ 20% (HR 0.60 [95% CI 0.49, 0.73]) or ≥ 30% (HR 0.55 [95% CI 0.45, 0.68]) had significantly longer PFS. Similar results were seen for the impact of ETS ≥ 20% (HR 0.47 [95% CI 0.38, 0.58]) or ≥ 30% (HR 0.48 [95% CI 0.38, 0.59]) on OS. In PRIME, the optimal ETS cut-off for prediction of improved OS outcomes was 32% (*p* < 0.0001).

#### PEAK

Overall, 154 patients were included in the ETS analyses; 106 patients (69%) achieved ETS ≥ 20% and 84 (55%) achieved ETS ≥ 30% (Rivera et al. 2017). Of the patients with ETS ≥ 20% and ETS ≥ 30%, respectively, 93 (88%) and 76 (90%) were subsequently confirmed as achieving a RECIST response (partial or complete), with the remainder having a best overall response of SD or PD. Twenty-three patients had a resection (R0 and/or R1) and ETS data, of these, 18 (78%) had ETS ≥ 20% and 15 (65%) had ETS ≥ 30%. Sixteen patients had R0 resections and ETS data, of these, 13 (81%) had ETS ≥ 20% and 12 (75%) had ETS ≥ 30%.

Compared with the bevacizumab plus mFOLFOX6 arm, more patients receiving panitumumab plus mFOLFOX6 had ETS ≥ 30% (64% vs. 45%, OR 1.99 [95% CI 0.99, 4.10]; *p* = 0.052) (Rivera et al. 2017). Similar observations were noted regarding the ETS ≥ 20% cut-off (75% vs. 62%, OR 1.67 [95% CI 0.78, 3.58]; *p* = 0.21). Factors associated with improved ETS in the final multiple regression model were panitumumab treatment (vs. bevacizumab), liver-only metastases (vs. liver + other or other only metastases) and WT *BRAF* status (vs. mutant) (Table 2b).

For those achieving ETS ≥ 20%, median PFS was 13.1 vs. 11.3 months in the panitumumab plus mFOLFOX6 vs. bevacizumab plus mFOLFOX6 group (HR 0.70 [95% CI 0.45, 1.08]; *p* = 0.11) (Rivera et al. 2017). Among those achieving ETS ≥ 30%, median PFS was 13.0 vs. 11.1 months, respectively (HR 0.74 [95% CI 0.45, 1.22]; *p* = 0.24). When treatment arms were combined, achievement of ETS ≥ 20% was associated with longer PFS (HR 0.55 [95% CI 0.37, 0.81]; *p* = 0.0029). Similar results were seen when combined data were analysed using the ≥ 30% ETS cut-off (HR 0.60 [95% CI 0.42, 0.87]; *p* = 0.0065). Likewise, irrespective of treatment received, patients achieving ETS ≥ 20% (HR 0.39 [95% CI 0.26, 0.59]; *p* < 0.0001) or ≥ 30% (HR 0.44 [95% CI 0.30, 0.65]; *p* < 0.0001) had longer OS. In PEAK, the optimal ETS cut-off for prediction of improved OS was 34% (*p* = 0.0006).

#### PLANET

Overall, 47 patients were included in the ETS analyses with 37 patients (79%) achieving ETS ≥ 20% (76 and 81% in the panitumumab + FOLFOX4 and panitumumab + FOLFIRI arms, respectively) (Abad et al. 2015). Thirty-one patients (66%) experienced ETS ≥ 30% (62 and 71% in the panitumumab + FOLFOX4 and panitumumab + FOLFIRI arms, respectively). As patient-level data were not available from PLANET, analyses of factors associated with ETS were not possible.

Overall resection rates (R0 and/or R1) were higher in patients achieving ETS ≥ 30% vs. < 30% (65% vs. 31%; *p* = 0.030); similar results were seen in those achieving ETS ≥ 20% vs. < 20% (59% vs. 30%; *p* = 0.19) (Abad et al. 2015). No data on R0 resections are currently available from PLANET.

Among those achieving ETS ≥ 20% and ETS ≥ 30%, PFS outcomes were similar between treatment arms (median PFS 14.2 vs. 14.9 months and 16.4 vs. 18.6 months in the panitumumab + FOLFOX4 and panitumumab + FOLFIRI arms, respectively) (Abad et al. 2015). When treatment arms were combined, achievement of ETS ≥ 20% was associated with longer PFS (HR 0.32 [95% CI 0.14, 0.70]; *p* = 0.005) and OS (HR 0.31 [95% CI 0.11, 0.83]; *p* = 0.020). Similarly, achievement of ETS ≥ 30% was associated with longer PFS (HR 0.41 [95% CI 0.21, 0.79]; *p* = 0.008) and OS (HR 0.28 [95% CI 0.10, 0.77]; *p* = 0.014) outcomes.

### Meta-analysis assessing the impact of early tumour shrinkage on outcome

**Fig. 1:**
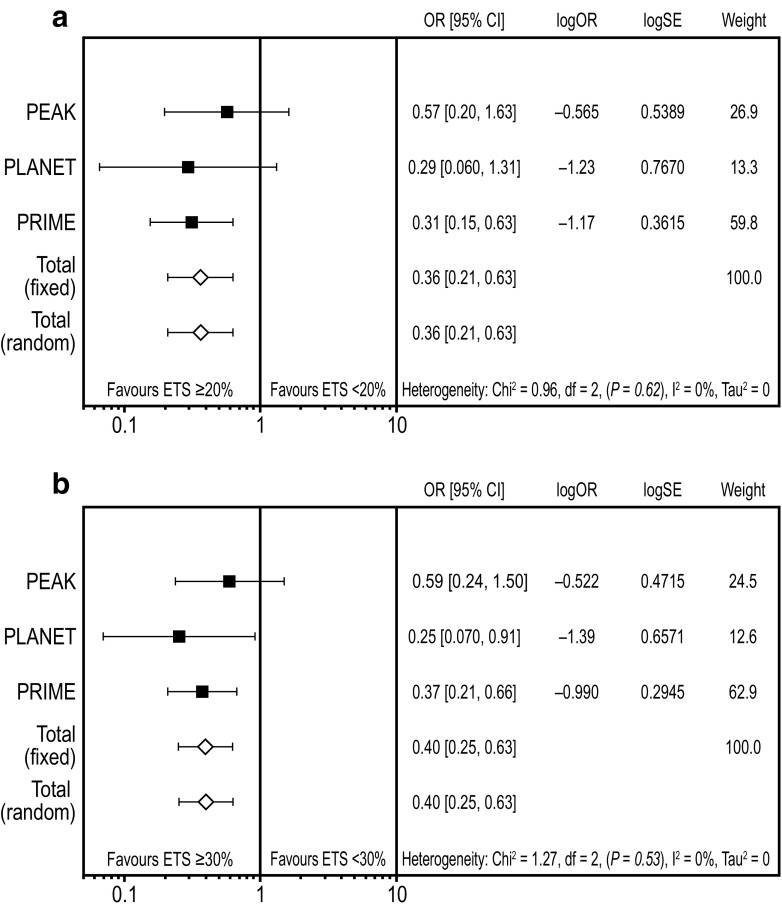
Meta-analysis assessing impact of early tumour shrinkage (**a** ≥ 20%; **b** ≥ 30%) on resection rates (*RAS* wild-type population) *CI* confidence interval, *ETS* early tumour shrinkage, *OR* odds ratio (for ETS ≥ 20%/ETS < 20% and ETS ≥ 30%/ETS < 30%, respectively), *SE* standard error weight is relative weight (%) from the fixed-effect models

Overall, 641 patients with *RAS* WT mCRC were evaluable for OS and ETS in these studies and so were included in the ETS meta-analyses (Rivera et al. 2016). This comprised 440, 154 and 47 patients from the PRIME, PEAK and PLANET studies, respectively. A meta-analysis of overall resection (R0 and/or R1) data favoured ETS ≥ 20% vs. ETS < 20% (OR 0.36 [95% CI 0.21, 0.63]; Fig. 1a) and ETS ≥ 30% vs. ETS < 30% (OR 0.40 [95% CI 0.25, 0.63]; Fig. 1b). Similarly, a meta-analysis of R0 resection data from PRIME and PEAK (data unavailable for PLANET) favoured ETS ≥ 20% vs. ETS < 20% (OR 0.31 [95% CI 0.15, 0.65]) and ETS ≥ 30% vs. ETS < 30% (OR 0.30 [95% CI 0.16, 0.55]). A meta-analysis of R0 resections performed in patients with liver-limited metastatic disease from these studies was also suggestive of a benefit for ETS ≥ 20% vs. < 20% (OR 0.87 [95% CI 0.31, 2.47]) and ETS ≥ 30% vs. < 30% (OR 0.51 [95% CI 0.21, 1.25]).

### Impact of early tumour shrinkage on progression-free and overall survival

**Fig. 2:**
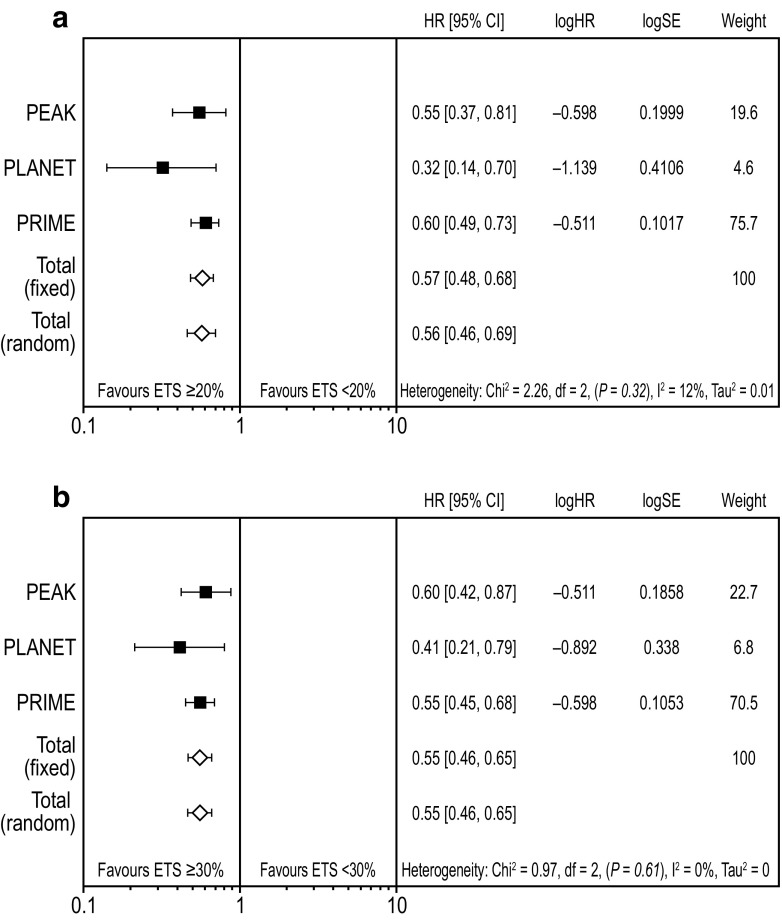
Meta-analysis assessing impact of early tumour shrinkage (**a** ≥ 20%; **b** 30%) on progression-free survival (*RAS* wild-type population) *CI* confidence interval, *ETS* early tumour shrinkage, *HR* hazard ratio, *SE* standard error weight is relative weight (%) from the fixed-effect models

**Fig. 3:**
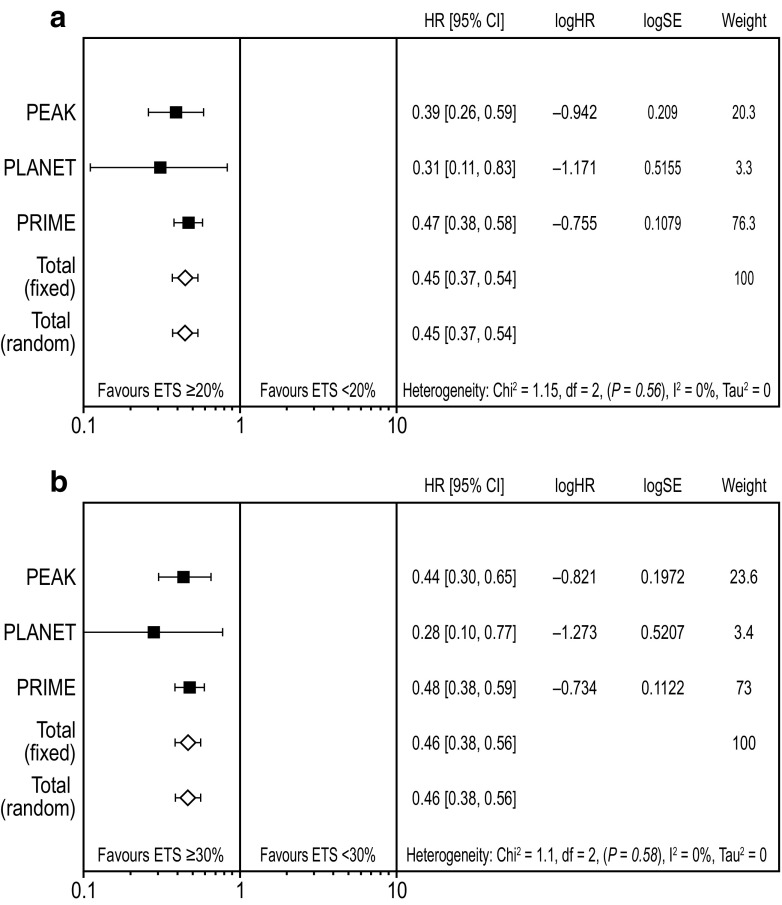
Meta-analysis assessing impact of early tumour shrinkage (**a** ≥ 20%; **b** ≥ 30%) on overall survival (*RAS* wild-type population) *CI* confidence interval, *ETS* early tumour shrinkage, *HR* hazard ratio, *SE* standard error weight is relative weight (%) from the fixed-effect models

Weighted meta-analysis results for PFS favoured ETS ≥ 20% vs. ETS < 20% (HR 0.57 [95% CI 0.48, 0.68]; Fig. 2a) (Rivera et al. 2016). Results were similar for ETS ≥ 30% vs. ETS < 30% (HR 0.55 [95% CI 0.46, 0.65]; Fig. 2b). Weighted meta-analysis results for OS also favoured ETS ≥ 20% vs. ETS < 20% (HR 0.45 [95% CI 0.37, 0.54]; Fig. 3a) and results were similar for the ETS ≥ 30% vs. ETS < 30% cut-offs (HR 0.46 [95% CI 0.38, 0.55]; Fig. 3b).

### Depth of response

Due to the nature of the data, a meta-analysis of DpR data was not possible; therefore, DpR results are presented by study.

#### PRIME

**Fig. 4:**
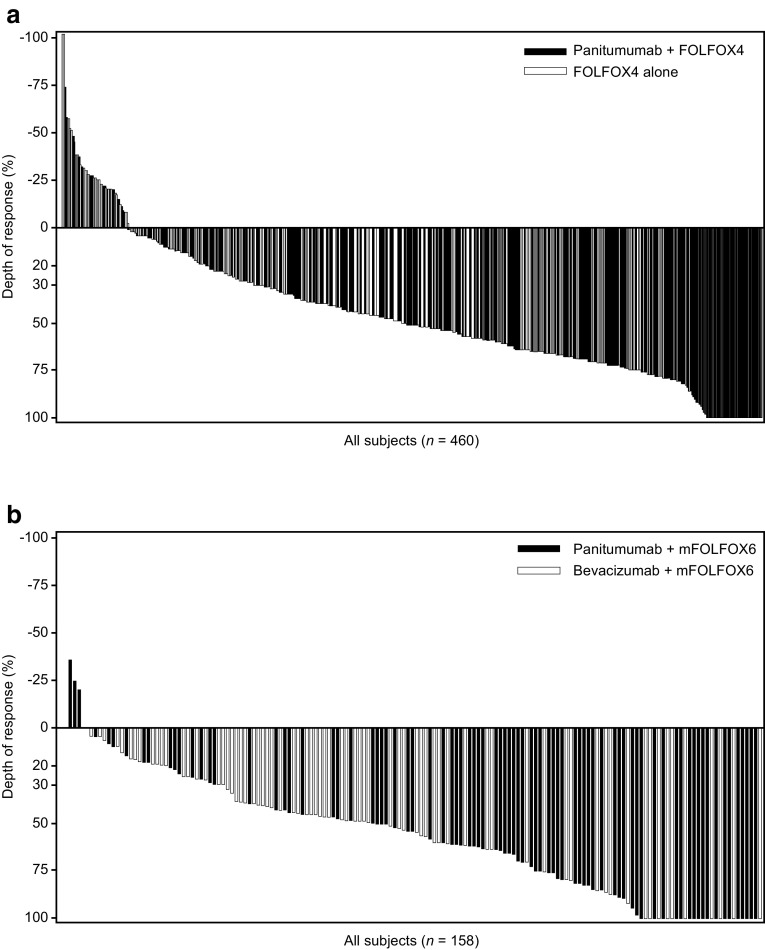
Waterfall plots showing distribution of depth of response in patients receiving panitumumab plus FOLFOX (blue bars) or comparator treatment (red bars) (**a** PRIME; **b** PEAK studies) (*RAS* wild-type population)

Overall, 460 patients with *RAS* WT mCRC were included in the analysis; median DpR was higher in patients receiving panitumumab plus FOLFOX4 vs. FOLFOX4 alone (54% vs. 46%; *p* = 0.0149) (Douillard et al. 2015). The distribution of DpR in the PRIME study (by treatment) is shown in Fig. 4a. Factors associated with improved DpR in the final multiple regression model were panitumumab treatment (vs. FOLFOX4 alone), liver-only metastatic disease (vs. liver + other or other only), WT *BRAF* status (vs. mutant) and an ECOG performance status of 0 or 1 (vs. 2) (Table 2a).

**Fig. 5:**
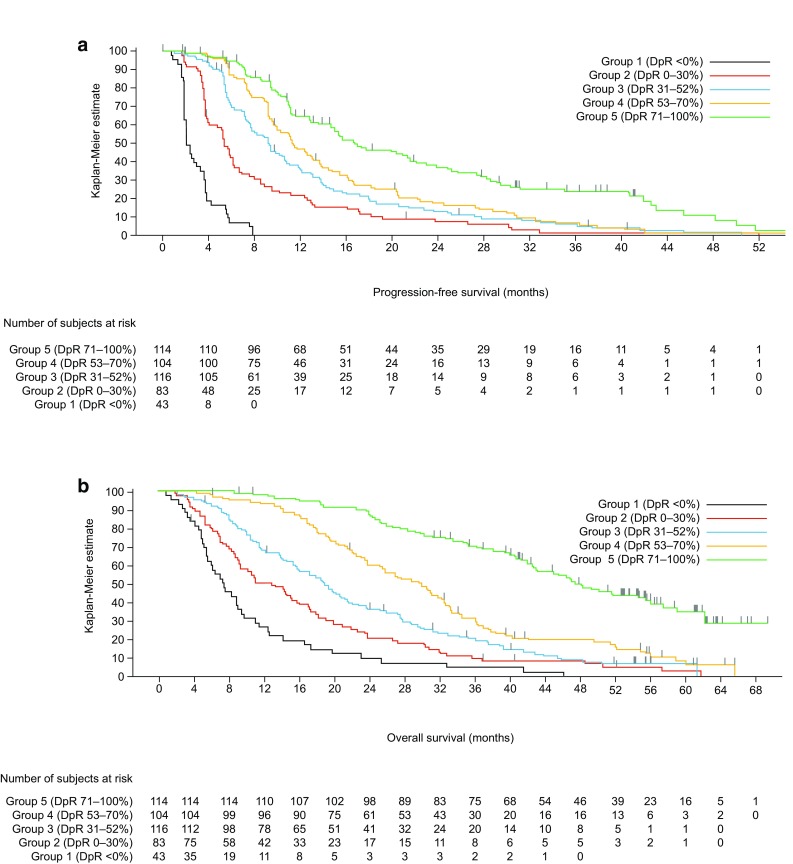
Impact of depth of response (DpR) on survival in the PRIME study (**a** progression-free survival; **b o**verall survival) (*RAS* wild-type population) censor indicated by vertical bar

Irrespective of treatment received, patients with deeper responses had longer PFS and OS (Table 3a, Fig. 5 and Supplementary Fig. S1A); median PFS and OS were shortest in those patients experiencing tumour growth (Group 1: DpR < 0%) (Siena et al. 2016). Median OS exceeded 48 months in those patients experiencing a DpR of 71–100%. DpR was also associated with PFS (*p* < 0.0001) and OS (*p* < 0.0001) when analysed as a continuous variable in a multiple Cox regression model (Table 2a). In PRIME, the optimal DpR cut-off for prediction of improved OS was 59%.

**Table 3:** Efficacy outcomes by depth of response category (a, PRIME; b, PEAK studies) (*RAS* wild-type population)

	DpR continuous (*n* = 460)	DpR category				
		Group 1 < 0% (*n* = 43)	Group 2 0–30% (*n* = 83)	Group 3 31–52% (*n* = 116)	Group 4 53–70% (*n* = 104)	Group 5 71–100% (*n* = 114)
PFS events, *n* (%)	–	43 (100.0)	79 (95.2)	107 (92.2)	96 (92.3)	87 (76.3)
Median PFS (95% CI), months	–	2.1 (1.9, 3.3)	5.4 (3.9, 6.1)	9.3 (7.6, 10.6)	11.3 (9.7, 13.7)	16.8 (14.6, 21.6)
HR (95% CI)^a^	0.78 (0.76, 0.81)	9.86 (6.7, 14.6)	1.70 (1.3, 2.3)	–	0.79 (0.60, 1.04)	0.46 (0.34, 0.61)
*P* value	< 0.0001	< 0.0001	0.0004	–	0.0915	< 0.0001
OS events, *n* (%)	–	42 (97.7)	79 (95.2)	103 (88.8)	90 (86.5)	66 (57.9)
Median OS (95% CI), months	–	7.5 (5.5, 9.4)	12.9 (9.2, 16.1)	18.9 (15.7, 21.4)	30.0 (23.8, 32.5)	48.1 (42.5, 56.0)
HR (95% CI)^a^	0.83 (0.81, 0.85)	2.92 (2.03, 4.19)	1.48 (1.10, 1.98)	–	0.63 (0.48, 0.84)	2.60 (0.19, 0.36)
*P* value	< 0.0001	< 0.0001	0.0092	–	0.0015	< 0.0001
Responders, *n* (%)	–	–	–	72 (62.1)	96 (92.3)	97 (85.1)
Median DoR (95% CI), months	–	–	–	7.6 (5.5, 9.5)	9.4 (7.9, 10.1)	13.9 (11.1, 19.3)
Any resection, *n* (%)	–	0 (0.0)	0 (0.0)	6 (5.2)	6 (5.8)	51 (44.7)
R0 resection, *n* (%)	–	0 (0.0)	0 (0.0)	5 (4.3)	1 (1.0)	39 (34.2)

	DpR continuous (*n* = 158)	DpR category				
		Group 1 <0% (*n* = 3)	Group 2 0–30% (*n* = 33)	Group 3 31–52% (*n* = 41)	Group 4 54–82% (*n* = 40)	Group 5 83–100% (*n* = 41)
PFS events, *n* (%)	–	3 (100.0)	30 (90.9)	39 (95.1)	39 (97.5)	28 (68.3)
Median PFS (95% CI), months	–	3.9 (1.8, 3.9)	7.6 (5.7, 11.6)	9.5 (7.2, 12.6)	13.0 (10.7, 15.1)	18.8 (13.2, 24.8)
HR (95% CI)^a^	0.80 (0.75, 0.85)	18.98 (5.0, 72.4)	1.54 (1.0, 2.5)	–	0.69 (0.44, 1.08)	0.27 (0.16, 0.45)
*P* value	< 0.0001	< 0.0001	0.0781	–	0.1007	< 0.0001
OS events, *n* (%)	–	2 (66.7)	29 (87.9)	37 (90.2)	29 (72.5)	9 (22.0)
Median OS (95% CI), months	–	15.0 (8.9, 21.2)	17.3 (11.1, 21.8)	23.9 (16.9, 28.9)	36.5 (26.0, 43.8)	63.0 (48.0, NE)
HR (95% CI)^a^	0.78 (0.73, 0.83)	2.62 (0.62, 11.05)	1.26 (0.77, 2.05)	–	0.49 (0.30, 0.80)	0.09 (0.04, 0.19)
*P* value	< 0.0001	0.1898	0.3543	–	0.0046	< 0.0001
Responders, *n* (%)	–	–	–	34 (82.9)	38 (95.0)	33 (80.5)
Median DoR (95% CI), months	–	–	–	7.9 (5.5, 9.2)	11.1 (8.4, 13.5)	17.0 (10.3, 23.2)
Any resection, *n* (%)	–	0 (0.0)	1 (3.0)	0 (0.0)	2 (5.0)	20 (48.8)
R0 resection, *n* (%)	–	0 (0.0)	1 (3.03)	0 (0.0)	0 (0.0)	15 (36.6)

*CI* confidence interval, *DpR* depth of response, *HR* hazard ratio, *NE* not evaluable, *OS* overall survival, *PFS* progression-free survival, *R0* complete resection

^a^Group HR compared with Group 3, continuous HR estimate is for the HR associated with a 10% difference in DpR

^b^Test for trend

In an analysis using the RECIST cut-off for response, patients achieving a DpR of ≥ 30% had longer PFS (median 11.9 vs. 3.8 months, HR 3.25 [95% CI 2.62, 4.04]; *p* < 0.0001) and OS (median 30.3 vs. 9.4 months, HR 3.24 [95% CI 2.59, 4.05]; *p* < 0.0001) compared with those achieving a DpR of < 30%. Similarly, patients achieving a DpR of ≥ 20% had longer PFS (median 11.5 vs. 3.7 months, HR 5.89 [95% CI 4.55, 7.62]; *p* < 0.0001) and OS (median 28.7 vs. 8.9 months, HR 3.40 [95% CI 2.67, 4.34]; *p* < 0.0001) compared with those achieving a DpR of < 20%. The greater the DpR, the longer the median DoR and the higher the overall and R0 resection rates; the proportion of patients experiencing a RECIST response was also greatest in the two highest DpR categories (Table 3a).

#### PEAK

Overall, 158 patients were included in the DpR analysis; median DpR was greater in the panitumumab plus mFOLFOX6 vs. bevacizumab plus mFOLFOX6 group (65% vs. 46%; *p* = 0.0018) (Rivera et al. 2017). The distribution of DpR in the PEAK study (by treatment) is shown in Fig. 4b. Factors associated with improved DpR in the final multiple regression model were panitumumab treatment (vs. bevacizumab), liver-only metastatic disease (vs. liver + other or other only metastases), WT *BRAF* status (vs. mutant) and age (decreased vs. increased, continuous variable) (Table 2b).

**Fig. 6:**
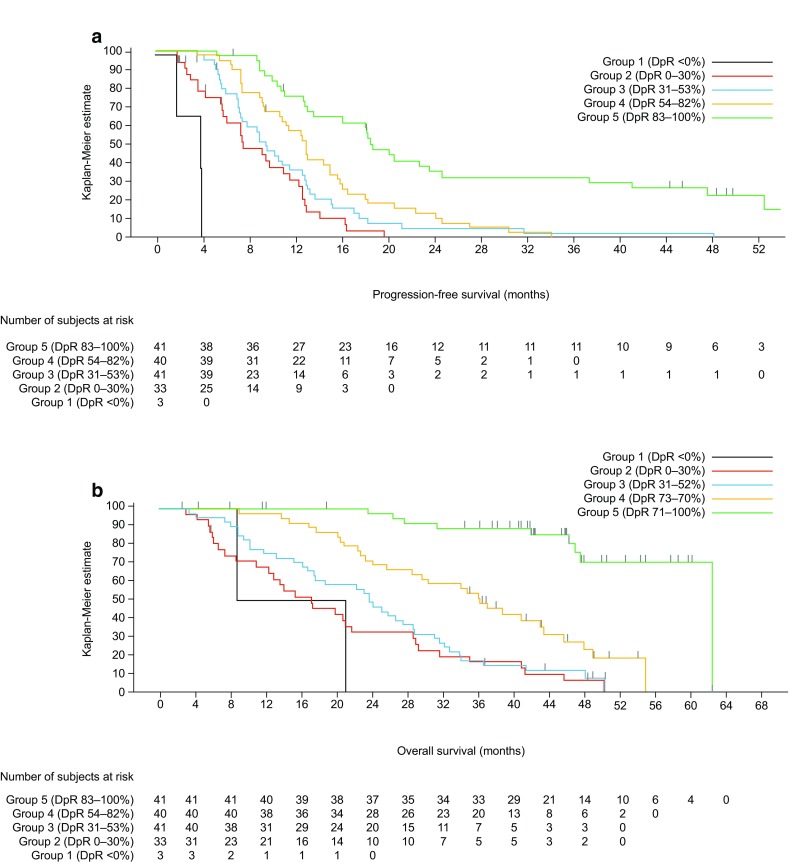
Impact of depth of response (DpR) on survival in the PEAK study (**a** progression-free survival; **b** overall survival) (*RAS* wild-type population) censor indicated by vertical bar

Patients with deeper responses had longer PFS and OS, irrespective of treatment (Table 3b, Fig. 6 and Supplementary Fig. S1B); median PFS and OS were shortest in those patients experiencing tumour growth (Group 1: DpR < 0%). Notably, median OS exceeded 60 months in patients experiencing the greatest DpR (Group 5: DpR of 83–100%). When analysed as a continuous variable in a multiple Cox regression model, DpR was also associated with PFS (*p* < 0.0001) and OS (*p* < 0.0001) (Table 3b). In PEAK, the optimal DpR cut-off for predicting improved OS was 70%.

Patients achieving a DpR of ≥ 30% had longer PFS (median 13.0 vs. 7.4 months, HR 2.80 [95% CI 1.86, 4.23]; *p* < 0.0001) and OS (median 37.4 vs. 17.3 months, HR 3.08 [95% CI 2.01, 4.71]; *p* < 0.0001) compared with those achieving a DpR of < 30%. Similarly, patients achieving a DpR of ≥ 20% had longer PFS (median 12.9 vs. 7.3 months, HR 2.88 [95% CI 1.77, 4.69]; *p* < 0.0001) and OS (median 34.4 vs. 21.0 months, HR 2.49 [95% CI 1.51, 4.11]; *p* < 0.0003) compared with those achieving a DpR of < 20%. Median DoR was longer and the resection rate higher in patients with the greatest DpR; the number of responders was also highest in the two top DpR categories (Table 3b).

#### PLANET

Fifty patients were included in the DpR analysis; median DpR was 48% overall and was similar in the panitumumab plus FOLFOX4 (47%) and panitumumab plus FOLFIRI (49%) groups (Abad et al. 2015). In patients with radiologically confirmed response (*n* = 24), median DpR was 67% overall (71% and 64% in the panitumumab + FOLFOX4 and panitumumab + FOLFIRI groups, respectively). As patient-level data were not available from PLANET, analyses of factors associated with DpR were not possible. No data are currently available from PLANET on the impact of DpR on outcome.

## Discussion

ETS offers the advantage of identifying responders and non-responders after 6–8 weeks of treatment, much earlier than is possible using older measures such as RECIST response. ETS, and also DpR, have previously been associated with long-term outcome in patients with mCRC (Cremolini et al. 2015; Heinemann et al. 2015). Here, we aimed to consolidate the available ETS and DpR data from first-line trials of panitumumab, some of which have only been reported in the form of congress abstracts (Abad et al. 2014, 2015; Rivera et al. 2016; Siena et al. 2016) or in part in full publications (Douillard et al. 2015; Rivera et al. 2017). We have also built on these data by reporting new exploratory analyses of the optimal ETS and DpR cut-offs to predict improved OS, factors associated with ETS and DpR, and the impact of these endpoints on response and resection, where possible. Taken together, the results of these analyses support an ETS and DpR benefit for panitumumab plus chemotherapy vs. chemotherapy alone or combined with bevacizumab. They are also in line with previous reports of an association between ETS (≥ 20% or ≥ 30% at week 8) and/or DpR during first-line treatment with favourable outcomes in patients with *RAS* WT mCRC, further supporting the use of these endpoints in the clinic. Furthermore, a recent exploratory analysis of a phase III trial comparing panitumumab plus best supportive care with best supportive care alone, suggested that ETS ≥ 0% during treatment may also be associated with PFS and OS benefits (Kim et al. 2017).

Individual study data from two randomised first-line panitumumab trials suggest that patients with *RAS* WT mCRC receiving panitumumab have higher rates of ETS than those receiving treatment without panitumumab (ETS ≥ 30%: PRIME 59% vs. 38% (Douillard et al. 2015); PEAK 64% vs. 45% (Rivera et al. 2017), respectively). In the new multiple regression analyses, factors associated with improved ETS that were consistent in both the PRIME and PEAK studies were panitumumab treatment, liver-only metastatic disease and WT *BRAF* status. ETS was associated with improved PFS and OS outcomes in all three first-line panitumumab studies (Abad et al. 2015; Douillard et al. 2015; Rivera et al. 2017) and also in the exploratory study-level meta-analysis (Rivera et al. 2016). The association of ETS with PFS and OS outcomes were similar irrespective of whether the data were analysed using the ≥ 20% or ≥ 30% cut-offs, suggesting that either cut-off can be used. These data support the value of ETS as a predictor for outcomes and are in line with those previously reported in first-line trials of cetuximab and bevacizumab (Modest et al. 2013; Cremolini et al. 2015; Giessen et al. 2013; Heinemann et al. 2015; Stintzing et al. 2016; Tsuji et al. 2016), and a meta-analysis of first-line data for chemotherapy ± targeted agents (Petrelli et al. 2015). Here we built on previous data for panitumumab by analysing the optimal ETS and DpR cut-offs for predicting improved OS. The ETS values reported here were similar in the PRIME and PEAK studies (32 and 34%, respectively), but were higher than the cut-off previously reported in the first-line bevacizumab TRIBE study (17%) (Cremolini et al. 2015). Although the ≥ 30% ETS cut-off is the same as that used to define a response in RECIST, the ETS measure differs in that it reports those achieving ≥ 30% shrinkage at a specific time point (week 8 here) and does not require that this is confirmed at a subsequent visit. ETS has the benefit that a result is gained more rapidly than for a best response based on RECIST and so can quickly identify early responders to treatment in the clinic. Non-responders can also be recognised sooner, thereby permitting an early switch to potentially more effective or better tolerated treatment.

Patients receiving panitumumab in these studies also had greater DpR compared with non-panitumumab-containing comparator arms in patients with *RAS* WT mCRC (Douillard et al. 2015; Rivera et al. 2017). In a new exploratory analysis assessing factors associated with DpR, the only factors that were consistently associated with improved DpR across the PRIME and PEAK studies were panitumumab treatment, liver-only metastatic disease and WT *BRAF* status. ECOG performance status and age were also associated in the PRIME and PEAK studies, respectively. Additional exploratory analyses from PRIME (Siena et al. 2016) and PEAK suggest that deeper responses are associated with longer PFS, OS and also improved DoR and higher resection rates. As might be expected, the vast majority of resections occurred in patients with the highest categories of DpR (71–100% in PRIME and 83–100% in PEAK). In line with previous reports, DpR was associated with PFS and OS, irrespective of treatment received (Nozawa et al. 2014; Cremolini et al. 2015; Heinemann et al. 2015; Stintzing et al. 2016; Tsuji et al. 2016). The optimal DpR cut-offs derived here for prediction of improved OS in the PRIME and PEAK studies were 59 and 70%, respectively, which are broadly in line with the cut-off reported in the TRIBE study (62%) (Cremolini et al. 2015). In PEAK, higher rates of ETS and greater median DpR were observed for panitumumab plus mFOLFOX6 vs. bevacizumab plus mFOLFOX6 (Rivera et al. 2017). Similar observations have been reported in the first-line FIRE-3 trial comparing cetuximab plus FOLFIRI vs. bevacizumab plus FOLFIRI (Stintzing et al. 2016). Interestingly, recent data from FIRE-3 suggest that ETS may also signal a subgroup of patients with right-sided mCRC who may benefit from treatment with an EGFRi plus chemotherapy (Holch et al. 2017). Taken together, these results suggest a potential benefit for EGFRis vs. bevacizumab for these response-related endpoints and could in part explain the improved OS also seen for these agents (Khattak et al. 2015; Heinemann et al. 2016).

In the clinic, achieving ETS and maximal DpR are likely to be of particular benefit to patients with symptomatic disease and those with potential to convert to resectable status. Consistent with this, in the studies analysed here, resections were mostly reported in patients who had experienced ≥ 20% or ≥ 30% ETS and in those with the greatest DpR. Achieving shrinkage early in potentially resectable patients could be important to permit resection as soon as possible, thereby avoiding potential liver toxicities and/or surgical complications due to prolonged treatment. Achieving these endpoints could also provide reassurance of likely treatment benefit and positive long-term survival outcomes.

Strengths of the present analyses include the fact that we included multiple panitumumab studies and a relatively large number of patients that the OS data from these studies are quite mature and that study-level meta-analyses were performed, where possible. We acknowledge there are, however, several limitations of these analyses—they were exploratory and retrospective in nature, there were differences in patient populations between studies, and the number of patients was limited in certain analyses. There is also a lack of clarity regarding the optimum ETS cut-off (20 or 30%) to use as definitions vary between studies. Furthermore, as we did not have access to patient-level data from all the studies, only a study-level meta-analysis of ETS could be performed and some of the ETS and DpR analyses were not possible for PLANET. There are also other factors unaccounted for in these analyses (e.g. primary tumour location (Yahagi et al. 2016), *BRAF* mutation status (Clarke and Kopetz 2015), etc.), which are also likely to impact on survival outcomes.

In conclusion, these exploratory analyses suggest that panitumumab plus chemotherapy may offer ETS and DpR benefits over chemotherapy alone or combined with bevacizumab in patients with *RAS* WT mCRC. Furthermore, ETS (≥ 20% or ≥ 30% at week 8) and DpR during first-line treatment are associated with favourable clinical outcomes.

## Electronic supplementary material

Below is the link to the electronic supplementary material.

Supplementary material 1 (PDF 402 kb)
