# Supplementary material for: Exploratory analyses assessing the impact of early tumour shrinkage and depth of response on survival outcomes in patients with RAS wild-type metastatic colorectal cancer receiving treatment in three randomised panitumumab trials
Source: J Cancer Res Clin Oncol. 2017 Oct 28;144(2):321–35. doi: 10.1007/s00432-017-2534-z (PMC5794806; doi:10.1007/s00432-017-2534-z)

Online supplementary material

Table S1 Baseline demographics and disease characteristics, by depth of response category (**a,** PRIME; **b,** PEAK studies) (*RAS* wild-type population)

a

	DpR category				
	**Group 1 <0% (*n* = 43)**	**Group 2 0–30% (*n* = 83)**	**Group 3 31–52% (*n* = 116)**	**Group 4 53–70% (*n* = 104)**	**Group 5 71–100% (*n* = 114)**
Study treatment, *n* (%)					
Panitumumab + FOLFOX4	17 (39.5)	40 (48.2)	57 (49.1)	53 (51.0)	69 (60.5)
FOLFOX4 alone	26 (60.5)	43 (51.8)	59 (50.9)	51 (49.0)	45 (39.5)
Male sex, *n* (%)	23 (53.5)	52 (62.7)	71 (61.2)	69 (66.3)	80 (70.2)
Age, years – median (range)	60 (32–79)	62 (27–80)	61 (32–79)	63 (37–82)	59 (27–77)
ECOG PS 0/1, *n* (%)	40 (93.0)	73 (88.0)	108 (93.1)	101 (97.1)	113 (99.1)
Prior adjuvant therapy, *n* (%)	8 (18.6)	21 (25.3)	16 (13.8)	16 (15.4)	11 (9.6)
Primary tumour diagnosis, *n* (%)					
Colon	31 (72.1)	54 (65.1)	80 (69.0)	55 (52.9)	77 (67.5)
Rectum	12 (27.9)	29 (34.9)	36 (31.0)	49 (47.1)	37 (32.5)
Side of disease, *n* (%)					
Left	25 (58.1)	45 (54.2)	73 (62.9)	79 (76.0)	87 (76.3)
Right	8 (18.6)	23 (27.7)	22 (19.0)	11 (10.6)	13 (11.4)
Unknown	10 (23.3)	15 (18.1)	21 (18.1)	14 (13.5)	14 (12.3)
BRAF status, *n* (%)					
Mutant	14 (32.6)	18 (21.7)	11 (9.5)	3 (2.9)	2 (1.8)
Wild-type	29 (67.4)	63 (75.9)	102 (87.9)	98 (94.2)	108 (94.7)
Unknown	0 (0.0)	2 (2.4)	3 (2.6)	3 (2.9)	4 (3.5)
Sites of metastases, *n* (%)					
Liver only	5 (11.6)	4 (4.8)	17 (14.7)	15 (14.4)	44 (38.6)
Liver + other	31 (72.1)	56 (67.5)	86 (74.1)	74 (71.2)	66 (57.9)
Other only	7 (16.3)	23 (27.7)	13 (11.2)	15 (14.4)	4 (3.5)

DpR depth of response, *ECOG PS* Eastern Cooperative Oncology Group performance status

b

	DpR category				
	**Group 1 <0% (*n* = 3)**	**Group 2 0–30% (*n* = 33)**	**Group 3 31–53% (*n* = 41)**	**Group 4 54–82% (*n* = 40)**	**Group 5 83–100% (*n* = 41)**
Study treatment, *n* (%)					
Panitumumab + mFOLFOX6	3 (100.0)	13 (39.4)	13 (31.7)	27 (67.5)	27 (65.9)
Bevacizumab + mFOLFOX6	0 (0.0)	20 (60.6)	28 (68.3)	13 (32.5)	14 (34.1)
Male sex, *n* (%)	2 (66.7)	24 (72.7)	29 (70.7)	27 (67.5)	24 (58.5)
Age, years – median (range)	59 (59–60)	63 (48–77)	62 (41–79)	61 (42–82)	58 (23–75)
ECOG PS 0/1, *n* (%)	3 (100.0)	33 (100.0)	41 (100.0)	40 (100)	41 (100)
Prior adjuvant therapy, *n* (%)	0 (0.0)	12 (36.4)	8 (19.4)	2 (5.0)	7 (17.1)
Primary tumour diagnosis, *n* (%)					
Colon	1 (33.3)	23 (69.7)	28 (68.3)	30 (75.0)	28 (68.3)
Rectum	2 (66.7)	10 (30.3)	13 (31.7)	10 (25.0)	13 (31.7)
Side of disease, *n* (%)					
Left	2 (66.7)	22 (66.7)	21 (51.2)	25 (62.5)	33 (80.5)
Right	1 (33.3)	9 (27.3)	10 (24.4)	7 (17.5)	5 (12.2)
Unknown	0 (0.0)	2 (6.1)	10 (24.4)	8 (20.0)	3 (7.3)
BRAF status, *n* (%)					
Mutant	1 (33.3)	4 (12.1)	0 (0.0)	7 (17.5)	1 (2.4)
Wild-type	2 (66.7)	29 (87.9)	41 (100.0)	33 (82.5)	40 (97.6)
Unknown	-	-	-	-	-
Sites of metastases, *n* (%)					
Liver only	1 (33.3)	7 (21.2)	7 (17.1)	9 (22.5)	19 (46.3)
Liver + other	1 (33.3)	11 (33.3)	19 (46.3)	23 (57.5)	15 (36.6)
Other only	1 (33.3)	15 (45.5)	15 (36.6)	8 (20.0)	7 (17.1)

DpR depth of response, *ECOG PS* Eastern Cooperative Oncology Group performance status

Fig. S1 Waterfall plots showing distribution of depth of response by overall survival status (blue bars: survived ≥24 months; red bars: survived <24 months) in patients receiving panitumumab plus FOLFOX or comparator treatment (**a,** PRIME; **b** PEAK studies) (*RAS* wild-type population)

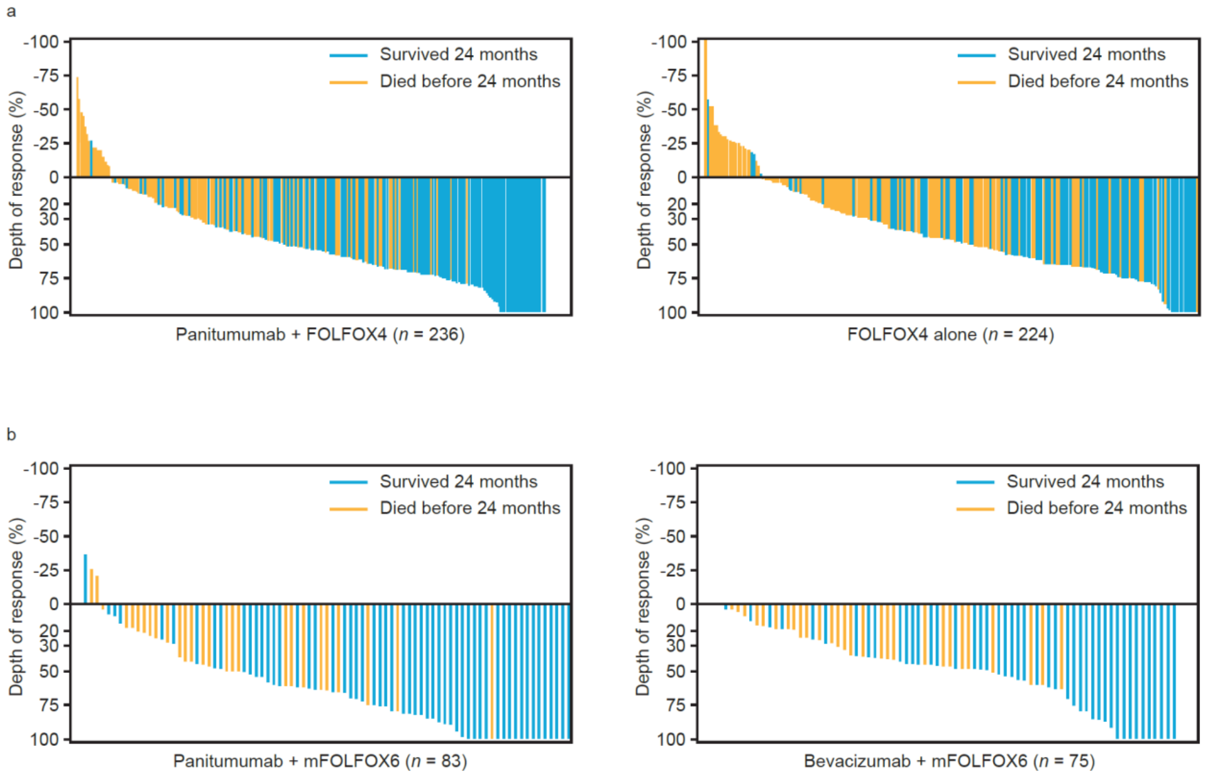

Supplement: Supplementary file 1 — Supplementary material 1 (PDF 402 kb) [file 432_2017_2534_MOESM1_ESM.pdf]
